# Comparative biomechanics of all-on-4 and vertical implant placement in asymmetrical mandibular: a finite element study

**DOI:** 10.1186/s12903-024-04186-w

**Published:** 2024-04-06

**Authors:** Xin Sun, Xiaodong Tang, Kangjie Cheng, Zhuoheng Xia, Yunfeng Liu, Fan Yang, Linhong Wang

**Affiliations:** 1 Center for Plastic and Reconstructive Surgery, Department of Stomatology, Zhejiang Provincial People’s Hospital, Affiliated People’s Hospital, Hangzhou Medical College, Hangzhou, Zhejiang China; 2 Department of Stomatology, Zhejiang Chinese Medical University, Hangzhou, Zhejiang China; 3 Tiantai County Hospital of Traditional Chinese Medicine, Taizhou, Zhejiang China; 4 College of Mechanical Engineering, Zhejiang University of Technology, Hangzhou, China

**Keywords:** All-on-4, All-on-5, Finite element analysis, Biomechanics, Cantilever

## Abstract

**Background:**

Clinical scenarios frequently present challenges when patients exhibit asymmetrical mandibular atrophy. The dilemma arises: should we adhere to the conventional All-on-4 technique, or should we contemplate placing vertically oriented implants on the side with sufficient bone mass? This study aims to employ three-dimensional finite element analysis to simulate and explore the biomechanical advantages of each approach.

**Methods:**

A finite element model, derived from computed tomography (CT) data, was utilized to simulate the nonhomogeneous features of the mandible. Three configurations—All-on-4, All-on-5-v and All-on-5-o were studied. Vertical and oblique forces of 200 N were applied unilaterally, and vertical force of 100 N was applied anteriorly to simulate different masticatory mechanisms. The maximum von Mises stresses on the implant and framework were recorded, as well as the maximum equivalent strain in the peri-implant bone.

**Results:**

The maximum stress values for all designs were located at the neck of the distal implant, and the maximum strains in the bone tissue were located around the distal implant. The All-on-5-o and All-on-5-v models exhibited reduced stresses and strains compared to All-on-4, highlighting the potential benefits of the additional implant. There were no considerable differences in stresses and strains between the All-on-5-o and All-on-5-v groups.

**Conclusions:**

With the presence of adequate bone volume on one side and severe atrophy of the contralateral bone, while the “All-on-4 concept” is a viable approach, vertical implant placement optimizes the transfer of forces between components and tissues.

## Introduction

The success of the All-on-4 technique has been widely established [1–4], particularly for cases involving severe atrophy of the mandibular bone, where there is insufficient vertical bone height in the posterior region. However, clinical scenarios frequently present themselves with patients exhibiting significant atrophy on one side of the mandible, while the other side possesses adequate alveolar bone height for implant placement. In such cases, a crucial decision arises: should the traditional All-on-4 technique be employed, or is it more advantageous to opt for conventional vertical implant placement on the side with sufficient bone volume?

The cantilever is currently considered to be the main factor influencing the success of implant-supported fixed prosthetic restorations, and it is widely recognized that the length of the cantilever of the upper restoration has a positive effect on the reduction of stress in the peri-implant bone [5]. Due to anatomical limitations, the classic All-on-4 may still produce a cantilever that increases the probability of mechanical complications in the restoration such as loosening of the abutment and screw and fracture of the upper bracket; moreover, the bending moment caused by the load applied to the cantilever can increase the force on the implant by 2-3 times, which is directly related to overloading of the peri-implant bone tissue [6, 7].

For patients with edentulous jaws, the alveolar bone heights on both sides of the jaw are often inconsistent. In clinical practice, such patients are often encountered: the absorption degree of the posterior dental area on both sides of the mandible is different, and the vertical bone volume of the posterior dental area on one side is sufficient, while the bone volume of the other side is insufficient, whether to choose the traditional All-on-4 technique or the option of adding vertical implant placement at the end of the cantilever on the side with sufficient bone is a dilemma that most implantologists will encounter in these cases. There have been no studies on the finite element aspects of performing 5-implant-supported fixed restorations in mandibular edentulous jaws with insufficient bone in the unilateral posterior region. And to date, the extent of the difference in load between vertically placed and angled implants in the case of distal bone abundance has been unclear [8–10].

This study compared the biomechanical aspects of vertical implant placement technique on the side with sufficient bone volume with the standard All-on-4 treatment concept by evaluating the stresses on the implants, and prosthetic components and strains in peri-implant bone in unilateral models of severely atrophied mandible treated with these techniques.

## Materials and methods

A finite element model of the edentulous mandible was constructed from computed tomography (CT) data, and 4 or 5 implants were placed in the mandible. Occlusal loading was simulated in the models to analyze the biomechanical behavior of the components and bone tissue.

### Design of the components

**Fig. 1:**
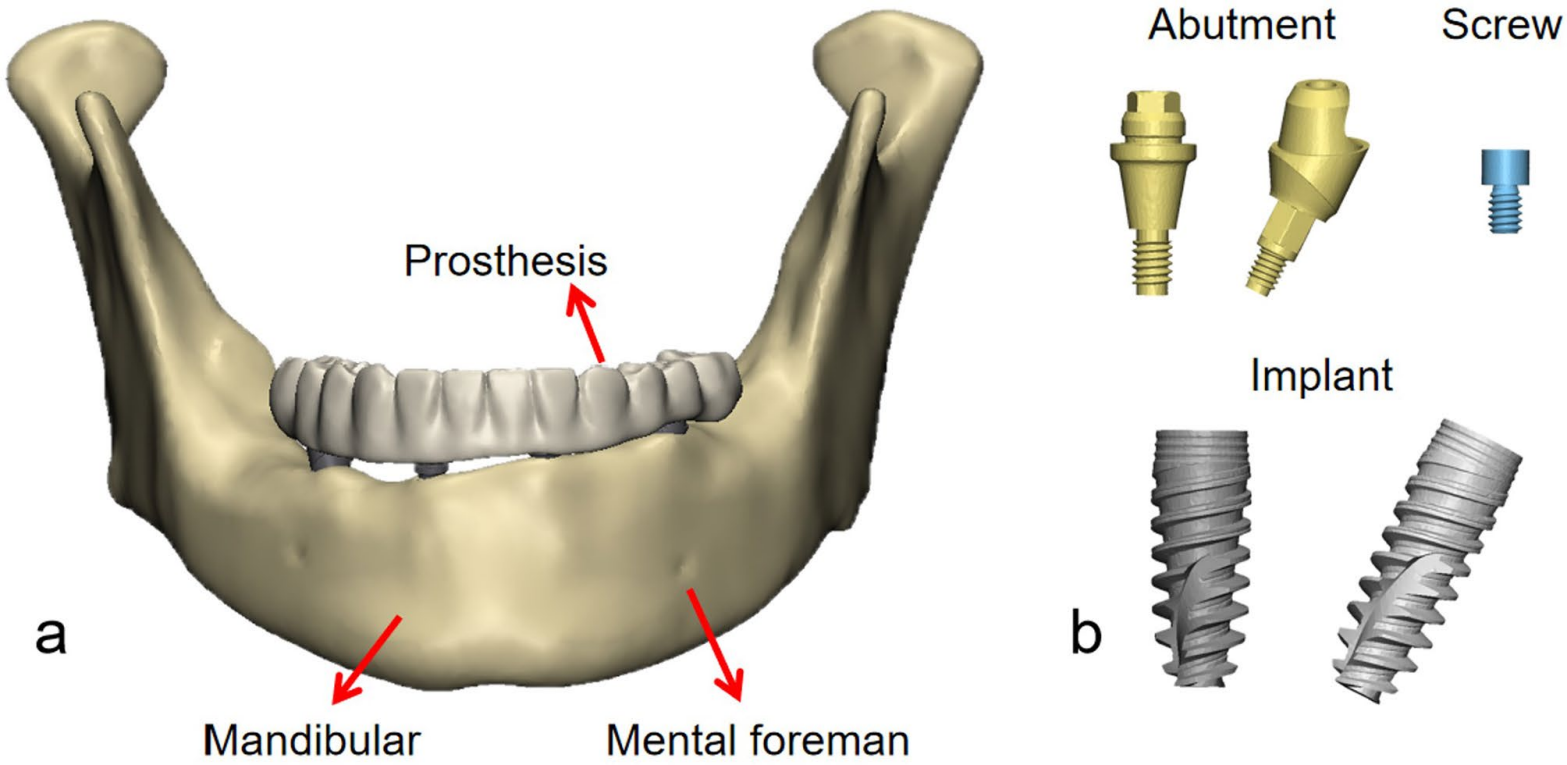
(**a**) 4-implant-supported model. The mandibular model reconstructed by CT showed varying degrees of bilateral atrophy of the alveolar bone, (**b**) Morphology and structure of implants, abutments and screws

In this study, a spiral CT scanner (Siemens, SOMATOM Definition AS128, Germany) was used to obtain CT data of the mandible of a 68-year-old healthy male volunteer. The scanning parameters were set to a voltage of 120 kV, a maximum tube current of 666 mA, and a layer thickness of 0.6 mm.The study protocol was approved by the Ethics Committee of Hospital (QT2023427), and written informed consent was obtained in advance. The CT data were imported in Digital Imaging and Communications in Medicine (DICOM) format into Mimics software (V19.0; Materialise), and a rough 3D contour of the mandible was obtained through thresholds and masks and exported as an Stereolithography (STL) file. The 3D solid model of the mandible showed different degrees of alveolar bone resorption in the posterior region bilaterally, with severe resorption of the alveolar bone behind the mental foramen on the right side, and adequate height and width of the alveolar ridge on the left side (Fig. 1a). Model of the dentition was obtained by scanning the denture in the patient’s mouth with a Computer Aided Design/Computer Aided Manufacturing (CAD/CAM) oral scanning machine (3 shape TRIOS), then exocad-Dental CAD (3.1 Rijeka) was used to trim and export to STL file.

**Fig. 2:**
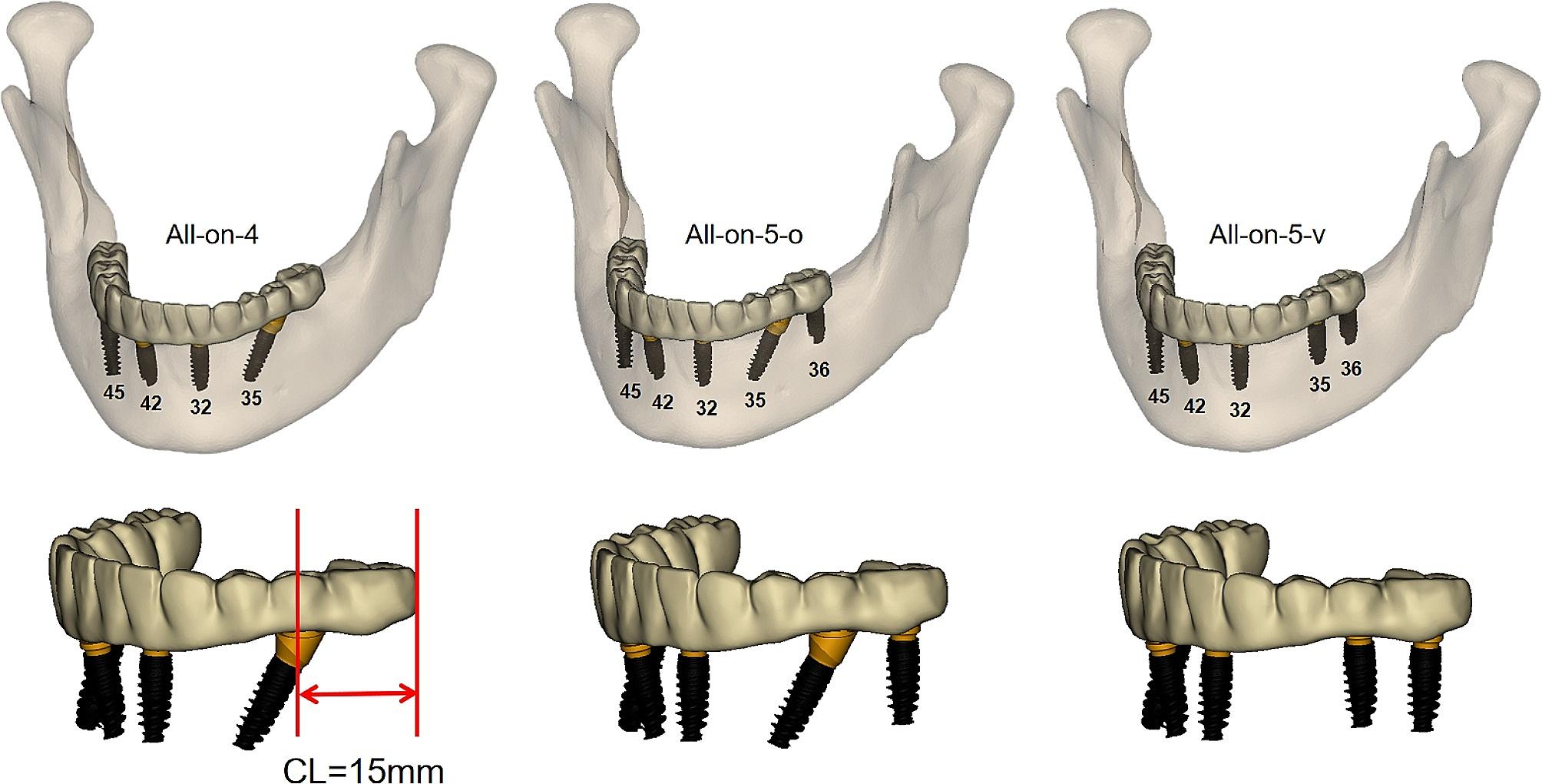
Three model designs. The cantilever length shown on the All-on-4 model was 15 mm

The models were imported into Geomagic Studio software (V13; Geomagic) for refinement, and the irregular burrs on its surface as well as the small unconnected areas inside were processed while not altering the morphology of the actual mandible and superstructure, to obtain complete and accurate 3D solid models in preparation for the next step of mesh delineation smoothly, and then exported in STL format. Finally, prosthesis and titanium framework models were obtained by extraction of the dentition, and the prosthesis was restored to the occlusal surface of the bilateral first molars (Fig. 2).

The implants in the model refer to the Nobel active system, and the implants were divided into three types according to length and position: the anterior dental implants were 3.5 mm × 11.5 mm, vertical implants in the posterior dental area were 4.3 mm × 11.5 mm, and oblique implants were 4.3 mm × 13 mm. Two abutment designs were employed, including a straight abutment with a height of 3.5 mm and an angle of 0°, and a 30° angled abutment with a height of 4.5 mm. Models were designed in SolidWorks (V2014; Dassault Systemes) based on the implant shape data, abutments, and screws (Fig. 1b).

### Grouping of models

Three groups were established: All-on-4, All-on-5-o, All-on-5-v (Fig. 2). The dental positions were numbered from the right to the left side of the mandible: 45, 42, 32, 35, 36, with 4 represents the right region, 3 represents the left region (Fig. 2). The All-on-4 group placed the terminal implants in positions 35 and 45 so that the cantilever length was set to 15 mm [11], and All-on-5-o and All-on-5-v groups added an implant at position 36 eliminating the left cantilever (Fig. 2).

### Meshing and metrics

Each set of models underwent importation into 3-matic software (V11.0) for the generation of tetrahedral elements. Prior to division, a specified mesh size was established: the mesh of the implant-bone contact surface and the contact surfaces of the abutment and framework underwent partial refinement, with the mesh size set to 0.2 mm, taking into consideration the mesh accuracy and the convergence speed of subsequent calculations.

The number of elements and nodes used by each model are shown in Table 1. The Abaqus software (V6.14; Dassault Systemes) was utilized to calculate von Mises stresses and equivalent strains. After convergence analysis, the error margin was less than 1%. Maximum von Mises stresses were recorded on implants and frameworks to describe the material deformation stress state [12]. Equivalent strains of peri-implant bone can be used to predict the bone remodeling nature as compared to bone resorption threshold limits [13–17]. Subsequently, the data were systematically gathered and color-coded for the purpose of comparative evaluation.

**Table 1:** Elements and nodes for each model

Model	Elements	Nodes
All-on-4 model	2,272,953	301,847
All-on-5-o model	2,343,983	313,764
All-on-5-v model	2,396,788	320,904

### Material properties

A three-dimensional model of the mandible with non-uniform material properties corresponding to the bone properties of different anatomical sites was established to improve the accuracy of the finite element models. The material properties of bone was determined from different radiodensity values of CT images [18]. The material properties (bone density and elastic modulus) of Hounsfield unit (*HU*) based model of the CT image were defined using Mimics software (V19.0, Materialise, Leuven, Belgium. The correlation between *HU* and bone density (ρ_0_) was derived from Eq. (1) [18].

1 $$\rho_{0}{\rm{ }} = 47 + {\rm{ }}1.122{\rm{ }} \times HU\left[ {kg/{m^3}} \right]$$

The elastic modulus (E) is related to the bone density (ρ_0_) as described in Eq. (2).

2 $$E = 0.63 \times \rho_{0}{^{1.35}}\left[ {MPa} \right]$$

**Fig. 3:**
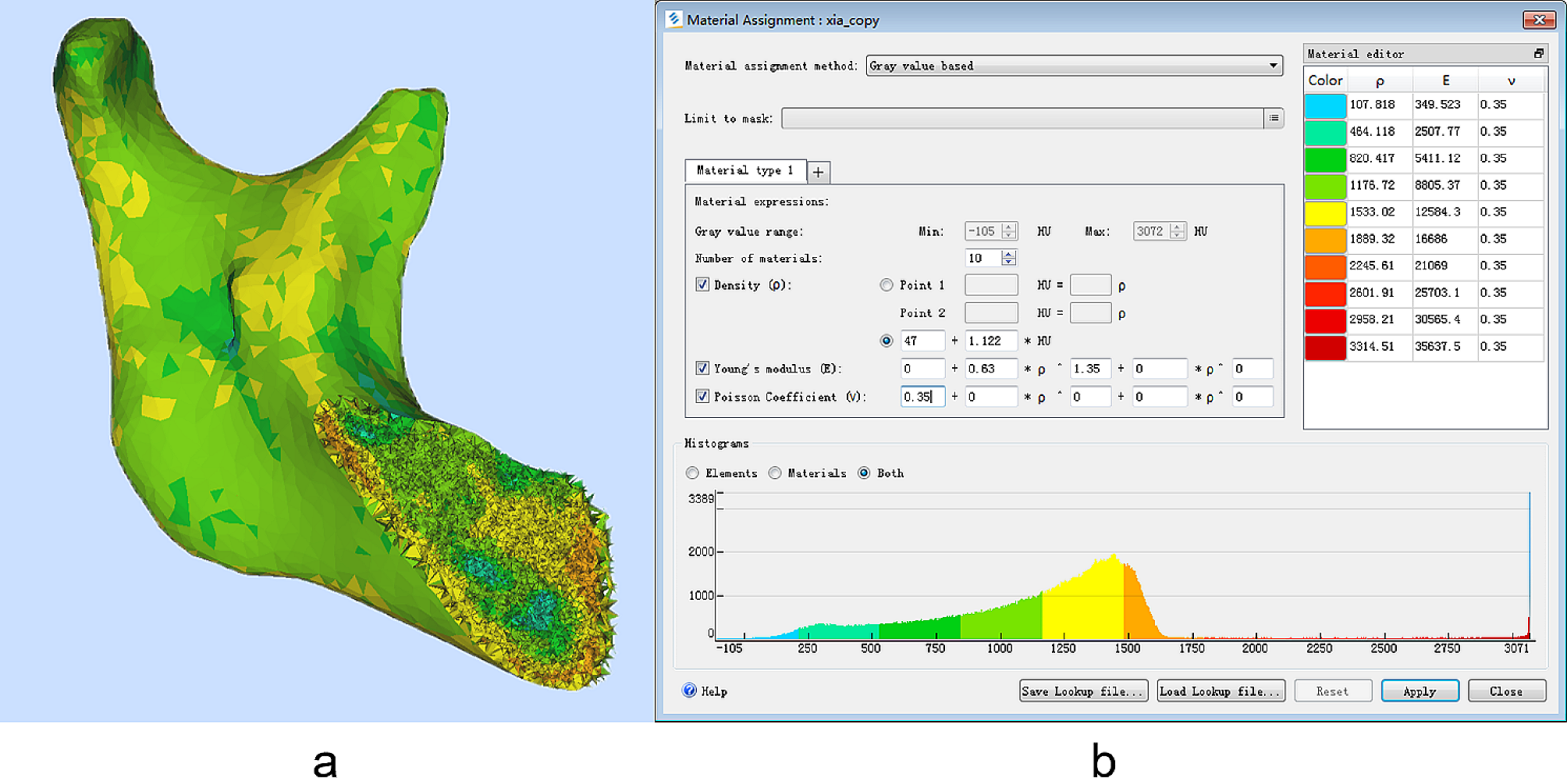
(**a**) Sagittal cross-sectional image of inhomogeneous mandible with different bone density mappings, (**b**) Material properties generation Interface

Figure 3a shows the color-coded mass density mapping of our model. Figure 3b shows the corresponding colors and mass densities and elastic moduli which were derived from Eqs. (1) and (2). The Poisson’s ratio was set as 0.35. Combined with Fig. 3a and b, it can be seen that the density of the mandible ranges from 107 to 1889 kg/m^3^, the elastic modulus ranges from 349 to 16,686 MPa, and the Poisson ratio is 0.3. The cortical bone is mainly yellow and orange, that is, the elastic modulus is between 12 and 17 GPa. Cancellous bone is mainly cyan and blue, that is, the elastic modulus is small. In addition, the mandible surface showed some cyan images, which is likely due to Mimics’ failure to accurately separate the mandible from soft tissue residues such as periosteum. After material attribute assignment, the mandibular tetrahedral mesh model was saved and exported in INP format. It can be seen from the results that the material properties assigned to the mandible are within a reasonable range [19], which verifies the rationality and validity of the derived linear equation between the apparent bone density and the *HU* scale and the logarithmic equation between the elastic modulus and the apparent bone density for constructing the material properties of the mandible.

The elastic modulus of acrylic and titanium is 3000 MPa and 112 GPa respectively, both parts made of pure titanium and prosthesis made of acrylic material have the Poisson’s ratio of 0.35 [20].

### Constraints and loading conditions

**Fig. 4:**
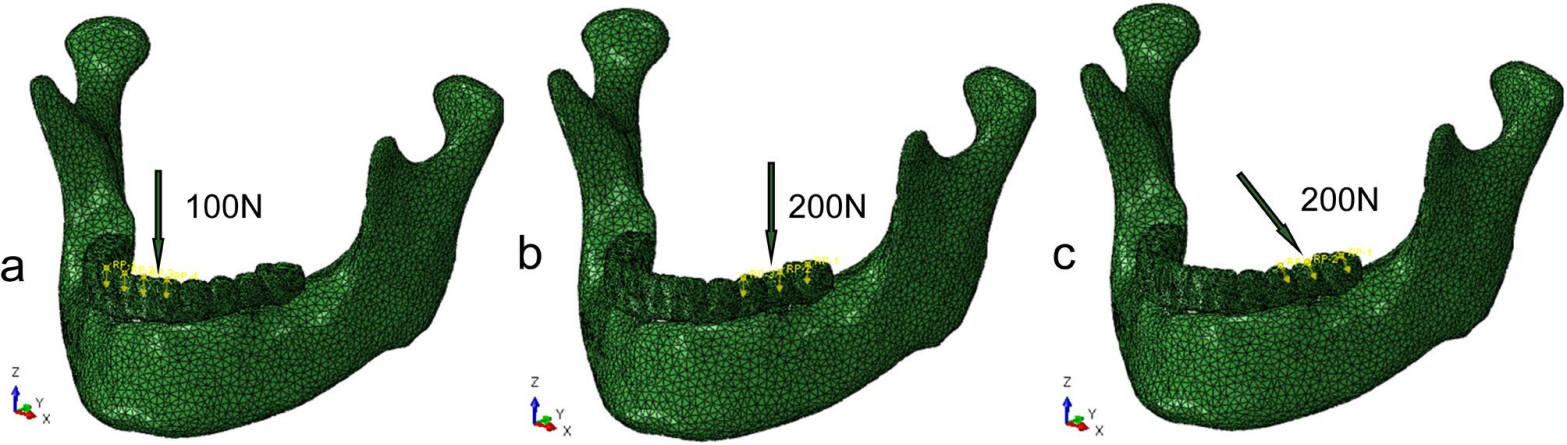
(**a**) Simulated vertical occlusal load of 100 N in the incisal region, (**b**) Simulated vertical occlusal load of 200 N in the left posterior region, (**c**) Simulated oblique occlusal load of 200 N in the left posterior region

To accurately simulate anatomically normal mandibular function, the bilateral condyles were assumed to be locked in the articular fossa and constrained to full degrees of freedom [21–23]. Materials were modeled with small deformations and linear elastic behavior. The implant-bone interface was configured as 100% contact, incorporating rigid restraints to prevent any relative sliding during application. Following the “All-on-4” concept, the average force applied to the premolar and molar regions in fixed prostheses approximated 200–300 N, and for incisors, it ranged from 100 to 300 N [24]. To simulate the clinical masticatory forces, each model used three loading patterns: (1) Vertical load in anterior teeth area (Load AV): 100 N vertical load in the incisors region (Fig. 4a); (2) Vertical load in posterior teeth area (Load PV): 200 N vertical load in the left posterior teeth region (Fig. 4b); (3) Oblique load in posterior teeth area (Load PO): 200 N oblique load in the left posterior teeth region with a 45°lingual-buccal tilt (Fig. 4c).

## Results

### Max von Mises stresses on the Dental Implants

**Fig. 5:**
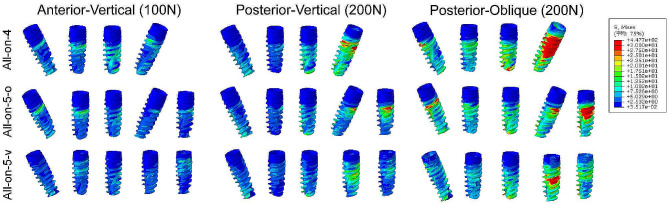
Distribution of von Mises stresses on implants under three different loads

Figure 5 shows the stresses distribution at the implant level under three different models. The stresses in all groups of implants were concentrated in the cervical region under the three loading patterns (Fig. 5). When force was applied to the anterior region, bilateral implants experience a uniform force, while applying force to the posterior region results in a larger red area (indicating high stress) for the last two implants near the loaded, with each implant experiencing an uneven force. The area of stress concentration on the implant at position 35 was largest in the All-on-4 model compared to the All-on-5 models for all three loads (Fig. 5). The maximum von Mises stresses were observed at the neck of the 35 implant in the All-on-4 model under load PV (215.0 MPa) and load PO (447.7 MPa) (Table 2). The maximum stresses on this implant were reduced by 38.6% in All-on-5-o and 35.2% in All-on-5-v compared to All-on-4 under load PV, with reductions of 41.1% and 46.3%, respectively, under load PO (Table 2). When force was applied in the anterior region, the maximum stresses were detected on the 45 implant compared to when force was applied in the posterior region, and similar maximum stress values were found across all three groups (Table 2).

As can be seen in Table 2, the stresses on the implant were related to the direction of loading, with the value of stresses under inclined loading being approximately twice as high as under vertical loading. In addition, the All-on-5-o group had similar stress levels to the All-on-5-v group.

**Table 2:** The Maximum von Mises stress values in implants (MPa)

Load	Model	45	42	32	35	36
Anterior-Vertical loading (100 N)	All-on-4	92.8	60.5	68.8	56.0	-
	All-on-5-o	97.1	58.6	52.6	22.1	33.6
	All-on-5-v	90.6	39.3	63.2	26.3	51.2
Posterior-Vertical loading (200 N)	All-on-4	26.1	33.3	65.4	215.0	-
	All-on-5-o	49.2	25.2	61.0	132.0	141.4
	All-on-5-v	86.6	41.4	48.5	139.4	102.1
Posterior-Oblique loading (200 N)	All-on-4	110.7	81.2	183.2	447.7	-
	All-on-5-o	141.9	95.5	209.0	263.5	434.4
	All-on-5-v	202.3	60.8	149.9	240.4	239.9

### Max von Mises stresses on the Frameworks

**Fig. 6:**
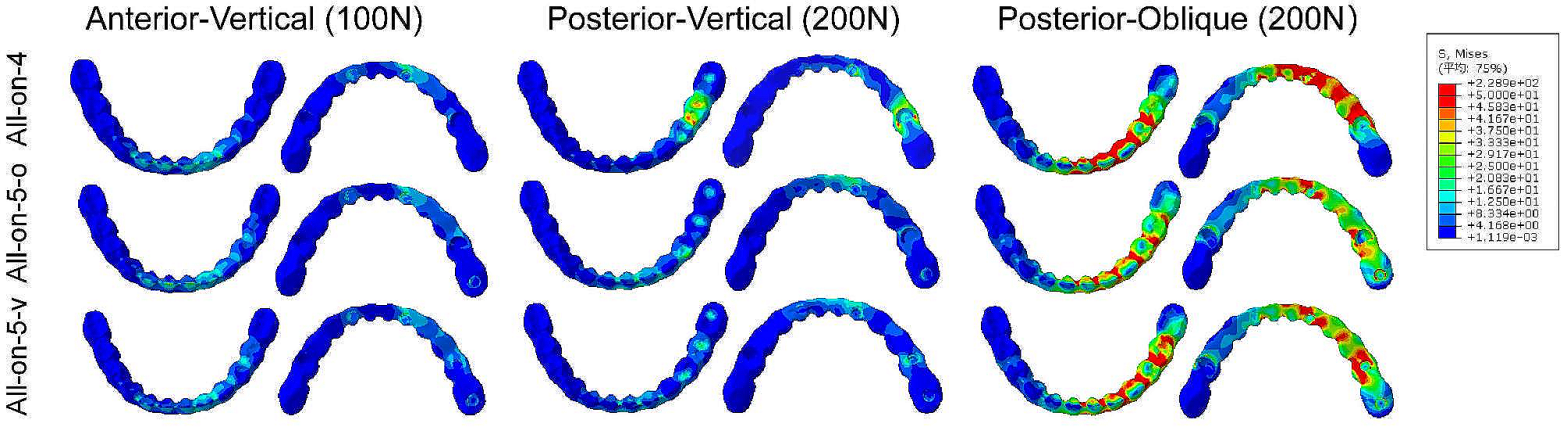
Distribution of von Mises stresses on framework under three different loads

The stress distribution on the framework showed a consistent trend in all three models, with higher stresses in the areas near the location of the applied force and lower stresses in the areas away from the applied force (Fig. 6). When the load was located in the posterior region, the red area detected in the All-on-4 group when subjected to a 45° angled load was the largest, whereas the other two groups were both reduced compared to the All-on-4 group (Fig. 6).

The stress values on the framework of the All-on-5-o model presented the lowest results when the force was applied in the posterior region (Table 3). However, the maximum von Mises stress values on the framework when the applied force was in anterior teeth were lowest in All-on-4 group (Table 3). The maximum von Mises stress values found on the frameworks under oblique loading were higher than vertical loading, about three to four times (Table 3).

**Table 3:** The Maximum von Mises stress values in framework (MPa)

Model	Anterior-Vertical loading (100 N)	Posterior-Vertical loading (200 N)	Posterior-Oblique loading (200 N)
All-on-4	33.1	89.3	213.8
All-on-5-o	61.2	40.2	161.5
All-on-5-v	37.6	58.7	228.9

### Equivalent strains in the peri-implant Bone

**Fig. 7:**
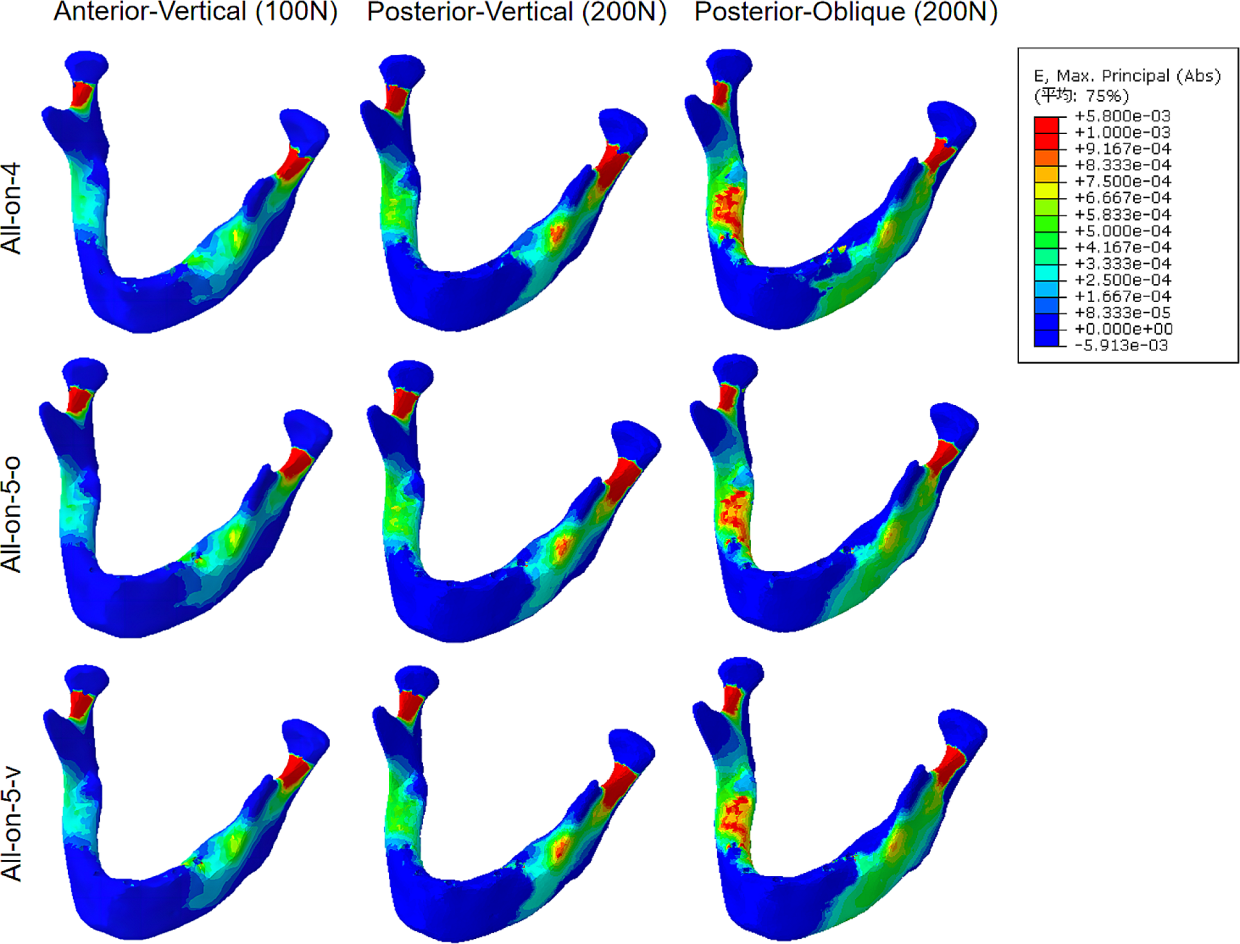
Equivalent strain distributions for three models under different loads

Figure 7; Table 4 summarize the maximum equivalent strains of the peri-implant bone for the three designs under the three loads. Higher strains were observed in all groups of models at the top of the alveolar ridge in the bilateral posterior region of the mandible under the anterior region load, while higher strains were observed in the buccal side of the left posterior region of the mandible as well as in the lingual side of the right posterior region of the mandible in all three models when the load was located in the posterior region (Fig. 7).

In comparison with the All-on-4 model, the peri-implant bone in the remaining two groups at position 35 showed reduced and more dispersed areas of strain (Fig. 7). Regardless of the load, the maximum strain values of each model bone were detected on the terminal implant, with the maximum equivalent strain of the bone tissue of the All-on-4 model being located on the bone around the peri-distal of the 35 implant, which was 0.76 × 10^3^ µε (load AV), 4.20 × 10^3^ µε (load PV), and 4.51 × 10^3^ µε (load PO) (Table 4), whereas placing the vertical implant placed at the left end of the two models, although the increased peri-implant strains around the vertical implant was greater, the maximum equivalent strain values of the peri-implant bone located at position 35 were reduced by 36.8% (All-on-5-o) and 73.7% (All-on-5-v), respectively, in comparison to the All-on-4 group under load AV, 75.7% (All-on-5-o) and 91.2% (All-on-5-v) reduction under load PV, and 74.1% (All-on-5-o) and 83.4% (All-on-5-v) reduction under load PO, respectively (Table 4).

The strains in the same designed model were greater under oblique loading as compared to vertical loading. In addition, the difference in strains on bone tissue between the All-on-5-o design and the All-on-5-v design was less (Fig. 7; Table 4).

**Table 4:** The Maximum equivalent strain values (10^3^µε) of bone around implants

Load	Model	45	42	32	35	36
Anterior-Vertical loading (100 N)	All-on-4	0.52	0.36	0.55	0.76	-
	All-on-5-o	0.48	0.36	0.56	0.48	3.71
	All-on-5-v	0.56	0.21	0.48	0.20	8.44
Posterior-Vertical loading (200 N)	All-on-4	0.60	0.37	1.16	4.20	-
	All-on-5-o	0.79	0.26	0.87	1.02	2.41
	All-on-5-v	0.91	0.21	0.61	0.37	5.68
Posterior-Oblique loading (200 N)	All-on-4	1.98	1.16	2.30	4.51	-
	All-on-5-o	3.75	0.93	2.39	1.17	3.31
	All-on-5-v	2.88	0.64	0.78	0.75	5.80

## Discussion

Previous findings affirm the well-documented success of the All-on-4 technique in cases of severe mandibular atrophy [1–4, 25, 26]. However, when faced with patients exhibiting unilateral atrophy and sufficient bone volume on the opposing side, our simulations suggest that conventional vertical implant placement may offer distinct biomechanical advantages. The three-dimensional finite element analysis provided insights into stress distribution patterns around implants and the surrounding tissues, aiding in the evaluation of the mechanical efficacy of each treatment modality. The decision between the traditional All-on-4 technique and vertical implant placement on the side with ample bone volume should be made on a case-by-case basis, considering the individual anatomical and clinical factors.

For the “All-on-4” operation on the mandible with edentulous mandible, whether the inclined implant at the end will be overloaded at the stress concentration site is a problem that we need to consider, and the influence of the stress on the surrounding bone tissue is also controversial. Some scholars [27, 28] believed that the cantilever design would increase the stress of the bone tissue around the distal implant, resulting in occlusal overload. Relatively speaking, the non-cantilever design of implant-supported fixation could better disperse the stress of the implant and the surrounding bone tissue [29, 30]. In this study, a case with severe unilateral mandibular alveolar bone atrophy was selected to extract the model, the advantage of this approach is that it accurately reflects the characteristics of the current clinical case. The differences in the biomechanical rows of the vertical implant placed at the cantilever on the side with sufficient bone were compared with the All-on-4 technique to provide biomechanical insights for making the most rational decisions when encountering such cases in the clinic.

The success of Three-dimensional finite element analysis (3D FEA) techniques has been reported to be related to the proportion of elements and nodes in the prepared mathematical model [31]. In this study, the All-on-4 model contained a total of 301,847 nodes and 2,272,953 elements, which is a sufficient number of nodes and elements to maximize the sensitivity of the analysis compared to similar studies [32, 33].

Compared to the All-on-4 technique, vertical implant placement with the lowest stresses and strains under three different forces represented the best-case condition in this study. The analysis results of all models showed that the maximum von Mises stresses of the implants were mainly concentrated in the neck of the distal implant on the loading side, which were consistent with the results of Sarrafpour [34] and Mahony et al [35]. Under different loads, the stress values of the implants and framework in both three models were lower than the yield strength of titanium (960 ∼ 1180MPa) [36]. In addition, the maximum stress values on the implants in all models were observed on the most distal implant in the All-on-4 group, whereas the All-on-5-o versus All-on-5-v models avoided the concentration of stress in the neck of the implant at position 35 due to the addition of the vertical implant placement, which may be attributed to the elimination of one side of the cantilever by the placement of the vertical implant, minimizing the negative biomechanical benefits.

Several studies have shown that long-term bone remodeling or bone resorption is a process of adaptation of biological systems to a mechanical state [37, 38]. The maximum strain values of the bone around the neck of the left distal-most implant were higher than the bone resorption threshold reported by Sugiura et al [14] (equivalent strain value of 3.6 × 10^3^µε)when subjected to a unilateral vertical or oblique force of 200 N by the conventional All-on-4 technique, and the maximum strains in the peri-implant bone at position 35 in the All-on-5-o and All-on-5-v groups were less than this value (Table 4). This shows that vertical implant placement technology can effectively reduce the risk of bone tissue absorption and better for long-term bone remodeling. It can be concluded that one-piece unilateral non-cantilevered restorations supported by a sufficient number of implants can optimize the transfer of force between different structures and tissues when there is sufficient bone volume on one side of the mandible.

In implant-supported fixed prosthetics, the clinical placement of implants was basically symmetrically distributed, and the technique of vertical implant placement proposed in this experiment resulted in an asymmetric distribution of implants in the mandible, which, according to the results, led to a more balanced distribution of stresses on all implants and strains on the surrounding bone tissues with no concentration of stresses on a single site. However, since there are fewer clinical efficacy studies related to the effect of symmetry on fixed restorations in edentulous jaws, more clinical studies should be conducted for further corroboration.

Some studies have found that the stresses under oblique load are significantly higher than that under vertical load, even up to 3.5 times [39]. The oblique load of 200 N was used in this study, and the stresses detected on each structure were significantly higher than the vertical load of 200 N. Therefore, the direction of the additional force also has a certain influence on the generation of stresses. The lateral force generated in the oblique load will form a lateral lever, and the resulting stresses are more likely to lead to the occurrence of related mechanical complications.

It is worth noting that an implant length of 11.5 mm was chosen for this study, mainly due to the sufficient bone volume on the left side of this mandibular model, and a longer implant would provide a larger implant-bone contact area, thus improving the implant’s stability. However, in practice, this may present some challenges, such as the need for more supportive bone volume and the possibility of compromising important anatomical structures. Therefore, although an 11.5 mm implant was selected, it may not be suitable for all clinical situations. In addition, in order to make the models more realistic and to improve the accuracy of the results, we set up the inhomogeneity of the jaw. However, in order to simplify the models and improve computational efficiency, this study simplified the partial design of the finite element model and set more idealized tissue and structural properties, such as neglecting the influence of the masticatory muscles, constraining the motion of the bilateral condyles and rostral processes, indeed, the mandible is surrounded by strong masticatory muscles attached to it, including the occlusal, temporal, intrapterygoid, and extrapterygoid muscles, which may have an impact on the biomechanical behavior of the mandible; Assuming that the mandible is linearly elastic and isotropic, in fact, the mandible has certain viscoelasticity and anisotropy [40]; As in other studies [41, 42] it was assumed that the implants were 100% osseointegrated with the bone. Although histologic studies have shown that osseointegration between the bone-implant interface has not been entirely materialized [43]. Our study was based on CT data of a patient with typical bone morphology characteristics. Therefore, the results of this study can only be used as a reference for patients with similar mandibular morphology. Also, in order to further confirm its validity and applicability, our results need to be validated in a larger group of patients.

Our study contributes valuable biomechanical insights to guide clinicians in making informed decisions tailored to the specific needs of patients facing such challenging scenarios. Future research and clinical trials are warranted to validate these simulated findings and further enhance our understanding of optimal treatment strategies for individuals with varying mandibular bone conditions.

## Conclusion

According to the findings of this study, unlike the conventional All-on-4 technique, the usage of vertical implant placement resulted in a reduction of stresses and strains on the implant and bone tissues in cases of adequate bone volume in the unilateral mandibular molar region and severe atrophy of the contralateral bone volume. Therefore, within the limitations of this study, it is believed that vertical implant placement could assume a more pivotal role in the rehabilitation of unilateral severely atrophic mandibles by optimizing the implant protocol design.

## Data Availability

All data are calculated by the software itself. The datasets used and/or analysed during the current study available from the corresponding author on reasonable request.

## Abbreviations

CT: Computed Tomography
DICOM: Digital Imaging and Communications in Medicine
STL: Stereolithography
CAD/CAM: Computer Aided Design/Computer Aided Manufacturing
3D FEA: Three-dimensional finite element analysis
